# Unraveling the Narrow Alley: A Case Report of Groove Pancreatitis

**DOI:** 10.7759/cureus.57683

**Published:** 2024-04-05

**Authors:** Mohamed A Ebrahim, Eli A Zaher, Parth Patel, Muhammad Sohaib Alvi, Shreyashi Khanal

**Affiliations:** 1 Internal Medicine, Ascension Saint Joseph Hospital, Chicago, USA

**Keywords:** whipples, alcohol, computed tomography, groove pancreatitis, pancreatitis

## Abstract

Groove pancreatitis, a rare subtype of chronic pancreatitis, predominantly affects middle-aged men with a history of alcohol abuse. We present a unique case of a 31-year-old female with minimal alcohol consumption. Imaging revealed characteristic findings consistent with groove pancreatitis. Despite its rarity in young females, clinical suspicion led to the appropriate diagnosis and conservative management, resulting in symptomatic resolution. This case underscores the importance of recognizing atypical presentations of groove pancreatitis, emphasizing the necessity of tailored diagnostic approaches, and highlighting the efficacy of conservative management in achieving favorable outcomes, particularly in non-typical demographics.

## Introduction

Groove pancreatitis, also known as paraduodenal pancreatitis, is a form of chronic pancreatitis involving the pancreas and duodenum. It is characterized by fibrotic scarring of the anatomic space between the pancreatic head, duodenum, and common bile duct called the pancreato-doudenal groove. Inflammation of the groove space only is classified as pure, while additional involvement of the pancreatic head classifies it as segmental. The prevalence of pure groove pancreatitis is 8.9%, and 15.5% for the segmental subtype. It mostly occurs in the 5th decade of life in men who abuse alcohol [1,2]. We present a unique case of groove pancreatitis in a young female with minimal alcohol use.

## Case presentation

A 31-year-old female with no significant past medical history presented to the emergency room with abdominal pain, nausea, and reduced appetite for two days. She described the pain as severe and sharp with associated radiation to the back. She denied having such symptoms in the past. She likewise denied any vomiting, diarrhea, fevers, or chills. The patient reported taking no medications at home. No excessive alcohol use was reported, with the last consumption being over two weeks prior to presentation. Surgical history was significant for a cholecystectomy seven years prior. Her family history was negative. 

**Figure 1:**
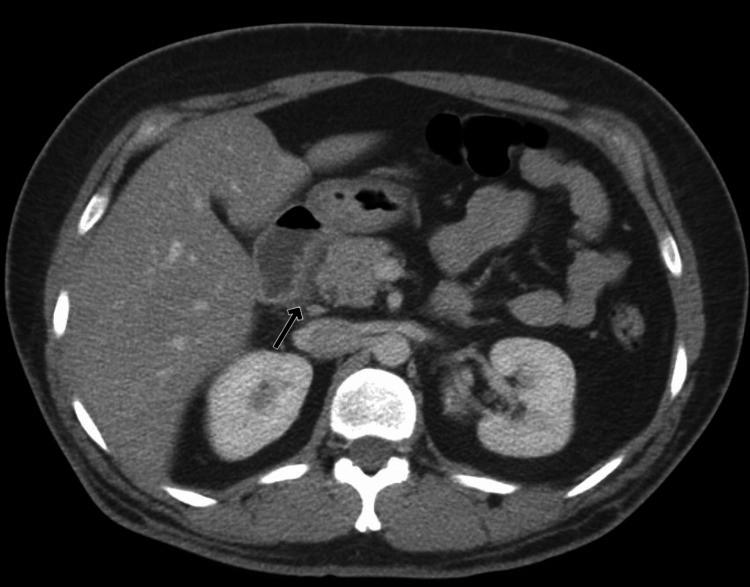
Axial CT abdomen with groove pancreatitis Arrow pointing towards fluid interdigitating between the descending portion of the duodenum and pancreas.

**Figure 2:**
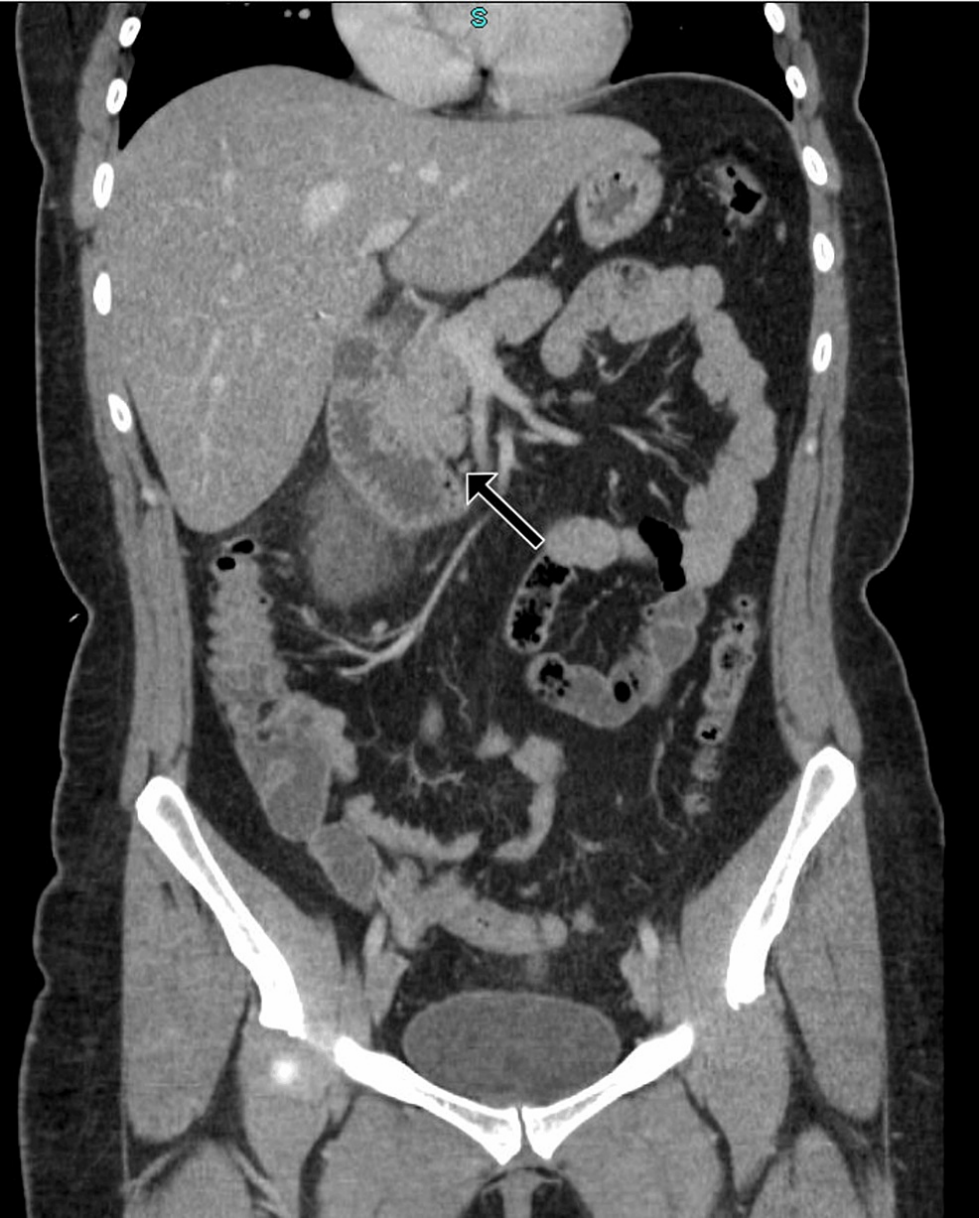
Coronal CT abdomen with groove pancreatitis Arrow pointing towards fluid interdigitating between the descending portion of the duodenum and pancreas. More fluid is identified inferior to the duodenum.

On admission, her vitals were within normal limits. Physical examination was consistent with epigastric tenderness without guarding but was otherwise unremarkable. Laboratory work-up was consistent with a mildly elevated lipase to 85 IU/L (reference range: 11- 82 IU/L). The rest of the laboratory work-up, including liver and lipid panels, was unremarkable. Computed tomography (CT) of the abdomen was consistent with groove pancreatitis (Figures 1-2). 

Given the above conditions, the patient was managed with intravenous fluid hydration, pain control, and pancreatic rest with avoidance of oral intake. Gastroenterology was consulted and an upper endoscopy was performed, which was unremarkable. The patient was negative for IgG subtype 4 antibody. Following symptomatic resolution with conservative management, she was discharged home in good health.

## Discussion

Groove pancreatitis, alternatively termed paraduodenal pancreatitis, represents a type of chronic pancreatitis affecting both the pancreas and the duodenum. It presents as a distinctive clinical entity, characterized by chronic segmental inflammation affecting both the duodenum and pancreas; it usually results from abnormal pancreatic secretions, which accumulate in the pancreatic head, leading to inflammation around the duodenum. The dorsal duct, known as the duct of Santorini, opens at the minor papilla. Inflammation causes alterations in the duodenum, potentially obstructing the minor papilla. Consequently, secretions are redirected to the main pancreatic duct, the duct of Wirsung, at an acute angle. This redirection results in incomplete drainage and retention of secretions in the pancreatic head, elevating intraductal pressure. Consequently, pancreatic fluid collection forms, and pancreatic secretions leak into the duodenal groove, prompting an acute inflammatory reaction [1,3]. Stole et al. categorized groove pancreatitis into two types: pure and segmental. In the pure form, inflammation is confined to the groove area alone, denoting a prevalence of 8.9%. Conversely, in the segmental form, inflammation extends extensively from the groove area into the head of the pancreas, with a prevalence of 15.5% [4]. 

The condition comprises a variation in clinical presentation, manifesting either acutely with tenderness in the upper abdomen leading to lingering discomfort, or chronically with symptoms such as postprandial abdominal pain, early satiety, nausea, vomiting, and weight loss, commonly associated with duodenal obstruction [5]. Jaundice occurs when the inflammation involves the common bile duct, mimicking the picture of pancreatic tumors, especially in older age [6]. Ultrasonography typically reveals a mass with increased echogenicity alongside thickening of the duodenal wall [7]. Abdominal CT scans aid in diagnosing groove pancreatitis and are consistent with histological findings. In the pure subtype, a hypodense laminar mass near the minor papilla and pancreatic head, indicative of scar tissue, is typically observed, accompanied by delayed contrast uptake due to reduced blood flow in fibrotic areas [8]. Cysts may also appear in the groove or duodenal wall. Conversely, the segmental form may exhibit a hypodense lesion near the duodenal wall, along with dilation of the main pancreatic duct in the body and tail of the pancreas.

Conservative treatment remains the cornerstone of therapy, comprising adherence to a well-balanced diet, use of pain relievers, pancreatic rest, and avoidance of alcohol and smoking [9]. While offering temporary alleviation, their effects are typically not enduring. Somatostatin analogs like octreotide have been utilized, exhibiting certain favorable outcomes [10]. In cases where symptomatic patients do not respond to conservative treatment or when there is uncertainty regarding the clinical diagnosis with lesions showing high suspicion of malignancy, surgery becomes the favored therapeutic approach. The preferred surgical intervention is pancreaticoduodenectomy, commonly known as the Whipple's procedure [11]. 

## Conclusions

Groove pancreatitis represents a rare form of chronic pancreatitis linked to ductal dysfunction. It's diagnosis often poses challenges, partly due to limited familiarity among healthcare providers, likely contributing to its low occurrence rates. Accurate identification of groove pancreatitis is crucial for determining the appropriate treatment approach, whether surgical intervention or medical management. While the correct diagnosis can avert unnecessary surgeries, thorough exclusion of pancreatic carcinoma is essential. While medical management is preferred, surgical intervention remains the standard of care in cases of obstructive symptoms or suspected malignancy. Therefore, it should be considered in the diagnostic process for patients presenting with lesions in the pancreatic head. 
